# Ivermectin Affects Neutrophil-Induced Inflammation through Inhibition of Hydroxylysine but Stimulation of Cathepsin G and Phenylalanine Secretion

**DOI:** 10.3390/biomedicines10123284

**Published:** 2022-12-19

**Authors:** Svetlana I. Galkina, Ekaterina A. Golenkina, Marina V. Serebryakova, Natalia V. Fedorova, Alexander L. Ksenofontov, Vladimir I. Stadnichuk, Galina F. Sud’ina

**Affiliations:** 1 A.N. Belozersky Institute of Physico-Chemical Biology, M.V. Lomonosov Moscow State University, Moscow 119991, Russia; 2 Physical Department, M.V. Lomonosov Moscow State University, Moscow 119991, Russia

**Keywords:** ivermectin, COVID-19 or coronavirus disease, neutrophil, adhesion, secretion, neutrophilic inflammation, hydroxylysine, phenylalanine, cathepsin G, angiotensin II (1–7)

## Abstract

The invasion and integrin-dependent adhesion of neutrophils to lung tissues and their secretion lead to the development of pneumonia in various pulmonary pathologies, including acute respiratory distress syndrome in coronavirus disease. We studied the effect of ivermectin, a possible therapeutic agent for inflammation and cancer, on integrin-dependent neutrophil adhesion to fibronectin and the concomitant secretion. Ivermectin did not affect the attachment of neutrophils to the substrate and the reactive oxygen species production but sharply inhibited the adhesion-induced release of hydroxylysine and stimulated the release of phenylalanine and cathepsin G. Hydroxylysine is a product of lysyl hydroxylase, which is overexpressed in tumor cells with an increased ability to invade and metastasize. The inhibition of hydroxylysine release by ivermectin, by analogy, may indicate the suppression of neutrophil invasion into tissue. The increase in the release of phenylalanine in our experiments coincided with the secretion of cathepsin G, which indicates the possible role of this enzyme in the cleavage of phenylalanine. What is the substrate in such a reaction is unknown. We demonstrated that exogenously added angiotensin II (1–8) can serve as a substrate for phenylalanine cleavage. Mass spectrometry revealed the formation of angiotensin II (1–7) in the secretion of neutrophils, which attached to fibronectin in the presence of ivermectin and exogenous angiotensin II (1–8), indicating a possible involvement of ivermectin in the inactivation of angiotensin II.

## 1. Introduction

Neutrophil tissue invasion and neutrophil-induced inflammation occurs with infections and in the absence of infection in some metabolic disorders such as reperfusion after ischemia and diabetes mellitus [1,2]. In the lungs, recruited neutrophils are involved in the development of pneumonia in cystic fibrosis, chronic obstructive pulmonary disease, and other pulmonary pathologies [3,4,5], including acute respiratory distress syndrome in coronavirus disease [6]. Ivermectin, an anti-parasitic drug with antiviral activity against several DNA and RNA viruses [7], is also proposed as a potential anti-inflammatory drug. Ivermectin may alleviate acute lung injury induced by lipopolysaccharide [8] and reduce in vivo coronavirus infection [9] in a mouse experimental models. Clinical studies on the effectiveness of ivermectin for the prevention and treatment of coronavirus disease are controversial. Some case series analysis demonstrates the ability of ivermectin to alleviate disease-associated severity and recovery rate [10,11]. Another analysis failed to demonstrate the efficacy of ivermectin in the treatment of COVID-19 [12,13]. The therapeutic effect of ivermectin is highly dependent on the phase of the disease in which the patient receives the drug [11]. Clinical trials have also revealed the high potential of ivermectin as an antitumor agent due to its ability to suppress tumor cell invasion into tissues leading to the formation of metastases [14]. We studied the effect of ivermectin on the function of neutrophils, typical representatives of innate immunity, which make a significant contribution to the development of inflammation.

Migration and infiltration of neutrophils into tissues are accompanied and provided by their own secretion of free amino acids and proteins. Our previous work has shown that neutrophil adhesion to fibronectin selectively stimulates an increase in the secretion of the free amino acid hydroxylysine, but does not alter the secretion of other amino acids [15,16]. Hydroxylysine is a product of lysyl hydroxylase (LH or procollagen-lysine, 2-oxoglutarate 5-dioxygenase, PLOD), which performs the post-translational modification of lysine residues of collagen in the rough endoplasmic reticulum. In tumor cells, LH is also secreted and functions in the extracellular space [17,18,19], thus affecting cell migration and adhesion via modification of the extracellular matrix [20]. A growing number of studies demonstrate that LH activity correlates with the ability of tumor cells to invade and metastasize [21,22,23,24,25,26,27]. By analogy with tumor cells, we hypothesize that the adhesion-induced increase in hydroxylysine release is associated with an increase in LH activity designed to promote neutrophil infiltration into tissues.

Neutrophils contain three types of secretory granules and secretory vesicles that differ in composition. Degranulation for each type of granule is triggered by different signaling pathways. Therefore, the composition of the secretion elicited by particular physiological or pharmacological agents varies greatly and is a characteristic property of these agents. Under control conditions, neutrophils secreted proteins of primary (MPO and lysozyme) and secondary granules (lactoferrin, NGAL), secretory vesicles (albumin), and cytosolic (S100A9 and S100A8) proteins [28]. The secretion of tertiary granule proteins, such as metalloproteinases, occurred upon neutrophil adhesion to fibronectin in the presence of insulin, 17ß-estradiol, or PMA [29,30]. Metalloproteinases play an important role in neutrophil migration [31,32,33,34]. The secretion of primary neutrophil granule components, such as cathepsin G, occurs during neutrophil adhesion to the substrate in the presence of the hormone glucagon or cytochalasin D, a microbial alkaloid that depolymerizes actin [28,30,35]. Cathepsin G participates in the regulation of inflammatory processes in several ways [36], including the modification of receptors on the cell surface [37] or due to possible involvement in the angiotensin–aldosterone pathway [38,39,40]. We studied the effect of ivermectin on the attachment of human neutrophils to substrates coated with fibronectin or fibrinogen and the accompanying secretion of proteins, free amino acids, and reactive oxygen species (ROS). Scanning electron microscopy has been used to study the morphology of neutrophils. Secreted proteins and amino acids were identified by mass spectrometry and amino acid analysis, respectively. The production of reactive oxygen species was judged by the fluorescence intensity of dichlorofluorescein (DCF).

## 2. Materials and Methods

### 2.1. Materials

Ficoll-Paque was obtained from Pharmacia (Uppsala, Sweden). Bicarbonate-free Hank’s balanced salt solution (HBSS), phosphate-buffered saline (PBS) and CaCl_2_-free Dulbecco’s PBS (D-PBS), thiol protease inhibitor E64, lipopolysaccharides from Salmonella enterica serotype typhimurium (LPS), and fibronectin from human plasma were obtained from Merck KGaA (Darmstadt, Germany). Ivermectin, N_ώ_-nitro-L-arginine methyl ester (L-NAME), tumor necrosis factor (TNF-α human), cytochalasin D, and human angiotensin II were obtained from Sigma (Steinheim, Germany). To stain the protein bands in the gel, we used Coomassie Brilliant Blue G-250 (Serva, Heidelberg, Germany). PMSF was from MP Biomedical (Irvine, CA, USA).Trypan blue from Honeywell Fluka (Charlotte, NC, USA) was used to demonstrate cell viability. Cells were fixed with glutaraldehyde from Ted Pella (Redding, CA, USA). Protein digestion in the gel was performed using trypsin from Promega (Madison, WI, USA). Carboxy-H_2_DCF-DA was obtained from Molecular Probe (Eugene, OR, USA). Eluent MCI Buffer L-8800-PH-1–4 and ninhydrin coloring solution kit for Hitachi 29,970,501 for analytical chromatography were from FUJIFILM Wako Chemicals GmbH (Richmond, VA, USA). Greiner CELLSTAR^®^ 6- and 96-well culture plates were from Greiner Bio-One GmbH (Kremsmünster, Austria).

### 2.2. Isolation of Neutrophils

The experiments with human blood were approved by the Bioethics Commission of M.V. Lomonosov Moscow State University, application # 6-h version 3, approved during the Bioethics Commission meeting # 131-days held on 31 May 2021. Neutrophils were isolated from the blood of healthy volunteers, who had given informed consent. The cell separation procedure was described in detail previously [16]. Briefly, after precipitation of erythrocytes with 3% Dextran T-500 solution, neutrophils were isolated from leukocyte-rich plasma by centrifugation through Ficoll-Paque at a density of 1.077 g/mL. The remaining erythrocytes were removed by hypotonic lysis followed by two washings in PBS. Neutrophils were suspended at a concentration of 10^7^ cells/mL in D-PBS containing 1 mg/mL glucose and stored at room temperature. The purity of the neutrophil suspension was 96–97%. The viability of neutrophils after isolation and after experiments was estimated with Trypan blue, which stains dead cells but did not penetrate viable cells. Neutrophils were incubated for 15 min at 37 °C with 0.5 mM Trypan blue in Hank’s solution and washed. The percent of stained cells, which was counted for 3000 total cells, did not exceed 1–2%.

### 2.3. Quantification of Neutrophil Attachment

First, 96-well culture plates were coated with fibronectin (5 µg/mL PBS) or fibrinogen (30 µg/mL PBS) for 2 h at a room temperature, followed by twice washing with warm PBS. Neutrophils were re-suspended in HBSS supplemented with 10 mM HEPES, pH 7.35 (HBSS/HEPES), to a concentration of 2 × 10^6^ cells/mL and shared at a rate of 2 × 10^5^ cells per well. After adding ivermectin (10 or 50 µM), the cells were incubated for 25 min at 37 °C in 5% CO_2_. At the end of the incubation time, free-floating or weakly attached cells were removed by gently washing the wells with warm PBS. The percentage of firmly attached cells was determined by measuring the absorption of 2,3-diaminophenazine, the colored product of MPO-catalyzed oxidation of o-phenylenediamine dihydrochloride by H_2_O_2_ [41]. The reaction mixture, containing H_2_O_2_ (4 mM) and 5.5 mM o-phenylenediamine dihydrochloride in permeabilizing buffer (67 mM Na_2_HPO_4_, 35 mM citric acid, and 0.1% Triton X-100), was added to the neutrophils for 5 min. The reaction was stopped by adding 1 M H_2_SO_4_. Absorption was measured at a wavelength of 490 nm. Standard cell dilutions were used for calibration.

### 2.4. Study of the Morphology of Attached Neutrophils by Scanning Electron Microscopy

Morphological changes were analyzed on the neutrophils attached to fibronectin-coated coverslips as previously described [35]. The procedure for substrate forming was identical to that described above for the culture plates. For attachment of the neutrophils, coated coverslips were placed into the wells of 6-well plates and 2 mL of cell suspension per probe (1.5 × 10^6^ cells/mL HBSS/HEPES) was applied. Incubation was carried out for 25 min at 37 °C under 5% CO_2_ after adding ivermectin (50 μM), L-NAME (200 μM), TNF (50 ng/mL), LPS (10 µg/mL) or their combinations, in accordance with the design of the experiment. Adherent cells were fixed by 2.5% glutaraldehyde in HBSS/HEPES supplemented with 5 mM EDTA and 0.5 mM PMSF and post-fixed in 1% osmium tetroxide in 0.1 M sodium cacodylate containing 0.1 M sucrose at pH 7.3. Then, the samples were dehydrated in a series of acetones (10–100%) and dried in a Balzer apparatus at the critical point with liquid CO_2_ as the transition liquid. The dried samples were sputtered with gold/palladium and examined on a Camscan S-2 scanning electron microscope at a voltage of 15 kV. ImageJ-win64 software was used to quantify the area occupied by neutrophils on the substrate.

### 2.5. Measuring of Reactive Oxygen Species (ROS) Formation

The effect of ivermectin on the formation of intracellular ROS was measured as intensity of the fluorescence of (DCF), the oxidation product of dichlorodihydrofluorescein diacetate (H_2_DCF-DA, Molecular probe, Molecular Probe (Eugene, OR, USA) [42]. Neutrophils were incubated with 5 μM carboxy-H_2_DCF-DA for 60 min in the dark at room temperature and washed. For subsequent visualization, the cell suspension (1 × 10^6^/mL HBSS/HEPES) was dispensed into fibrinogen-coated confocal dishes, incubated under control conditions or in the presence of ivermectin or PMA (positive control) for 25 min at 37 °C under 5% CO_2_. After incubation, the samples were fixed with 4% paraformaldehyde solution for 10 min at room temperature in the dark, and examined with a Zeiss Axiovert 200 M fluorescence microscope. To quantify the effect of ivermectin on the ROS production, H_2_DCF-loaded cells were plated onto fibronectin-coated 96-well plates (1 × 10^6^/mL) and incubated according to the experimental protocol at 37 °C in 5% CO_2_ under control conditions or in the presence of 10 or 50 µM ivermectin. Changes in fluorescence intensity upon emission of 538 nm and excitation of 485 nm were monitored at 5 min intervals on a ClarioStar fluorescence microplate reader (BMG).

### 2.6. Adhesion of Neutrophils to the Fibronectin-Coated Culture Plates and Sampling of Extracellular Medium for Amino Acid and Protein Analysis

First, 6-well culture plates were coated with fibronectin during 2 h incubation with fibronectin solution (5 µg/mL) at room temperature and washed. Neutrophils (3 × 10^6^ cells/mL in HBSS containing 10 mM HEPES at pH 7.35) were added to each well and incubated for 25 min at 37 °C in a 5% CO_2_ incubator under control conditions or in the presence of ivermectin (50 μM). Upon completion of the incubation, samples of the extracellular medium were taken from each well and a mixture of inhibitors was immediately added to each sample. The final concentration of the inhibitors in the medium was: 5 mM EDTA, an inhibitor of metalloproteinase; 200 µM PMSF, an inhibitor of serine proteinases; 10 µM E64, an inhibitor of cysteine proteinases; and 0.025% sodium azide, a myeloperoxidase inhibitor. Then, neutrophils not attached to the substrate were removed from the samples of the extracellular medium by centrifugation (5 min at 400× *g*).

### 2.7. Preparation of Samples for Amino Acid Analysis

For amino acid analysis, extracellular medium from three identical wells was pooled and concentrated using a Centrivap Concentrator Labconco (Kansas City, MO, USA). The proteins were then precipitated with sulfosalicylic acid (4.4%). The precipitate was removed by centrifugation for 30 min at 18,000× *g*. Before amino acid analysis, supernatant was further purified by centrifugation through Vivaspin 500 Membrane 3000 PES MWCO ultrafilters (Sartorius, Goettingen, Germany).

### 2.8. Amino Acid Analysis

The amino acid composition of the extracellular medium after the removal of proteins and the preliminary purification was quantified as described previously [16]. The amino acid analyzer L-8800 (Hitachi, Tokyo, Japan) was used in standard mode according to the manufacturer’s user manual (Hitachi High-Technologies Corporation, Japan, 1998). An ion-exchange column 2622SC (PH) (Hitachi, Tokyo, Japan, P/N 855–3508, 4.6 × 60 mm), with a step gradient of four sodium-acetate buffers at a flow rate of 0.4 mL/min at 57 °C, was used for amino acid separation. The MultiChrom program for Windows (OOO Ampersand, Moscow, Russia) was used for data processing.

### 2.9. Extraction and Separation Proteins of Extracellular Medium

To determine the protein composition of the neutrophil secretion, samples of extracellular medium from 6 identical wells were pooled. Proteins were extracted with Folch’s mixture (chloroform: methanol 2:1, *v*/*v*). Our previous data showed that the chloroform phases contained almost all of the detected proteins [28]. The chloroform phases were collected and, after evaporation of the chloroform, the proteins were separated by one-dimensional sodium dodecyl sulfate electrophoresis under non-reduced conditions on a 15% polyacrylamide gel in the Mini-PROTEAN 3 Cell (Bio-Rad, Berkeley, CA, USA). Separated proteins were stained with 0.22% Coomassie Brilliant Blue G-250.

### 2.10. Mass Spectrometry Identification of Proteins

The MALDI-ToF-ToF mass spectrometer Ultraflextreme (Bruker, Karlsruhe, Germany) was used for the MALDI-MS identification of proteins separated by electrophoresis as previously described [28]. From each protein band, gel pieces were excised, washed, dehydrated, and subjected to in-gel digestion with trypsin. The peptides obtained as a result of hydrolysis were extracted with 0.5% trifluoroacetic acid. For mass spectrometric analysis, aliquots of samples were mixed on a steel target with 2,5-dihydroxybenzoic acid (30 mg/mL in 30% acetonitrile, 0.5% trifluoroacetic acid). The [MH]^+^ molecular ions were measured in reflector mode. The accuracy of the monoisotopic mass peak measurement was within 30 ppm. Mascot 2.5.01 software (http://www.matrixscience.com, accessed on 3 January 2021) and the Swissprot mammalian protein database were used to search for peptide fingerprints. Protein matches were considered significant (*p* < 0.05) if the score was >68.

### 2.11. The Effect of Angiotensin II on the Composition of Peptides in the Extracellular Medium of Neutrophils

Neutrophils were attached to fibronectin for 90 min in the presence of 50 μM angiotensin II alone (1–8) or in II in combination with 50 μM ivermectin or 10 μg/mL cytochalasin. Samples of the extracellular medium were taken and non-attached neutrophils were removed from it by centrifugation. Samples of the extracellular medium were additionally incubated for 120 min at 37 °C. After that, aliquots were taken from each sample and subjected to mass spectrometric analysis.

### 2.12. Statistics

Each experiment was carried out at least 3 times using the blood of different individuals. GraphPad Prism 7 software from GraphPad (San Diego, CA, USA) was used for experimental data processing. Results are presented as mean ± SEM. One-way ANOVA was used for result processing, namely either Dunnett’s (for adhesion and ROS generation quantification data) or Tukey’s multiply comparison test (for data on measuring the area occupied by neutrophils on the substrate). Free amino acid secretion data were processed by two-way ANOVA with Tukey’s multiple comparison test. Significant (*p* ≤ 0.05) differences between the experimental groups are indicated as exact *p*-values on the graphs.

## 3. Results

### 3.1. The Effect of Ivermectin on Neutrophil Morphology and Attachment to the Fibronectin-Coated Substrata

**Figure 1:**
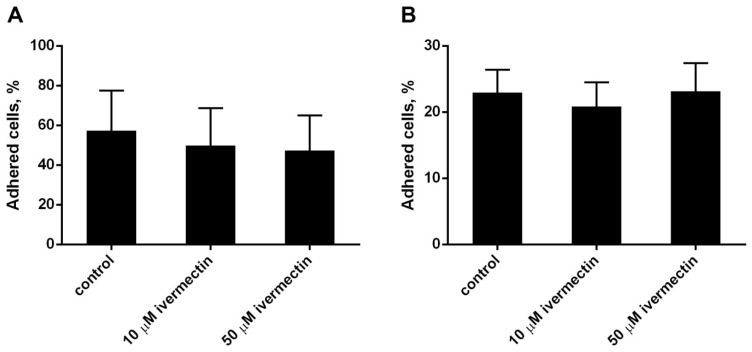
Attachment of neutrophils to fibrinogen- or fibronectin-coated substrata ((**A**,**B**), respectively). Neutrophils (2 × 10^6^ cells/mL) were incubated for 25 min at 37 °C, 5% CO_2_ in control conditions or in the presence of ivermectin. Shown are the percentages (means ± SEM, *n* = 3) of firmly attached cells from the total number of cells in the sample.

The effect of ivermectin on the attachment of neutrophils to the substrate was studied by a method based on measuring the absorption of 2,3-diaminophenazine, the colored product of the MPO-catalyzed oxidation of o-phenylenediamine dihydrochloride (OPD) H_2_O_2_. Ivermectin, which is widely used as an anthelmintic in animals and humans, has a relatively low toxicity across species [43,44]. We compared the effect of ivermectin on neutrophil adhesion to a substrate coated with the extracellular matrix proteins, such as fibronectin or fibrinogen. Neutrophils attach better to fibronectin than to fibrinogen (Figure 1A,B). Morphologically, the cells differed little. In both cases, ivermectin in the range of 10–50 μM had practically no effect on the attachment of neutrophils to the substrate compared to neutrophils that were attached to the same substrate under control conditions. In further experiments, fibronectin was used as a substrate for studying integrin-dependent cell adhesion.

**Figure 2:**
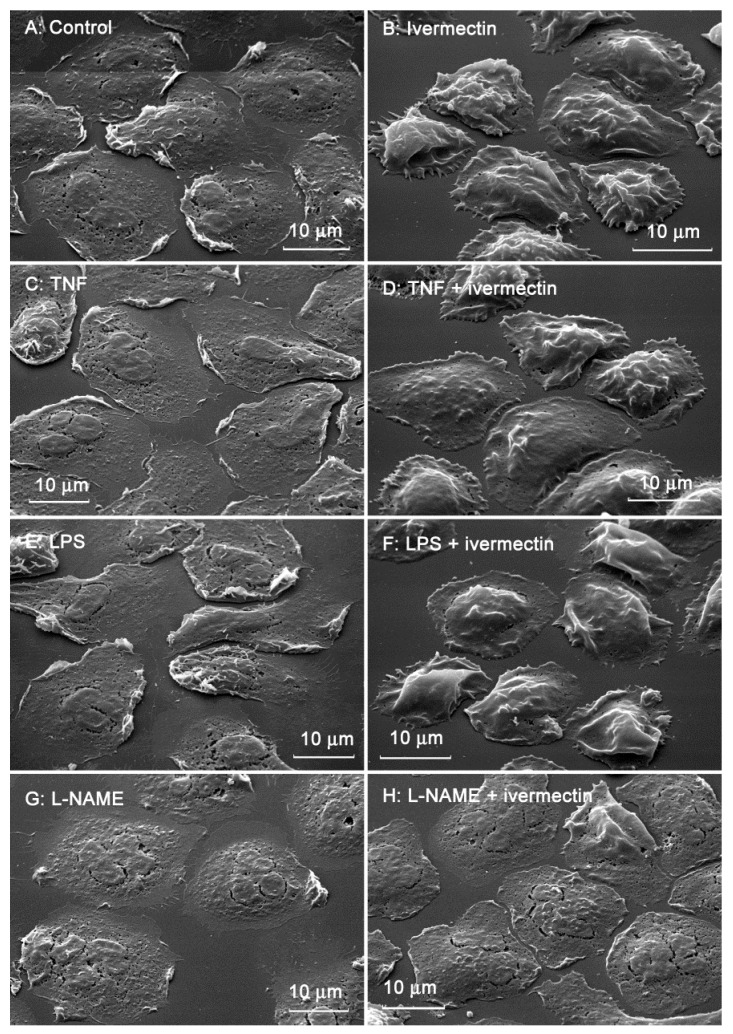
Scanning electron images of human neutrophils that were adhered to fibronectin-coated substrates for 25 min under control conditions (**A**) or in the presence of 50 µM ivermectin (**B**); in the presence of 50 nM TNF alone (**C**) or in combination with 50 µM ivermectin (**D**); in the presence of 1 µg/mL LPS alone (**E**) or in combination with 50 µM ivermectin (**F**); in the presence 200 µM L-NAME alone (**G**) or in combination with 50 µM ivermectin (**H**). The figures show typical images observed in three independent experiments.

The morphology of neutrophils attached to the fibronectin-coated substrates was assessed using scanning electron microscopy. Control neutrophils had spread morphology and a relatively smooth surface (Figure 2A). Neutrophils that attached to fibronectin in the presence of ivermectin were less spread and had a specific folded surface (Figure 2B). TNF (Figure 2C,D), a potent neutrophil-adhesion promoter, or LPS (Figure 2E,F), a potent stimulator of neutrophil activity, did not alter ivermectin-induced neutrophil folds. NAME, an inhibitor of nitric oxide (NO) synthesis, reversed the effect of ivermectin. L-NAME caused the spreading of ivermectin-treated neutrophils and the straightening of folds on the cell surface (Figure 2G,H).

**Figure 3:**
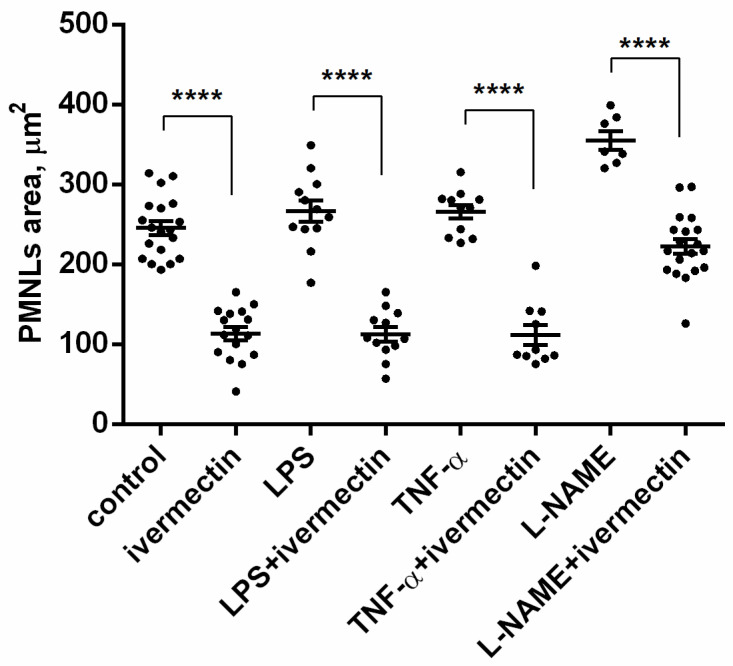
Area occupied by neutrophils that were adhered for 25 min to a fibronectin-coated substrate for 25 min under control conditions or in the presence of 50 µM ivermectin alone or in combination with 50 nM TNF, 1 µg/mL LPS, or 200 µM L-NAME. **** *p* < 0.0001, as shown one-way ANOVA, Tukey’s multiple comparison test.

The area occupied by neutrophils attached to fibronectin-coated coverslips on a substrate was measured on scanning electron images using ImageJ-win64 software. The area occupied by ivermectin-treated neutrophils was two times less than the area of control cells (Figure 3). The presence of TNF or LPS had no significant effect on the spreading of control or ivermectin-treated neutrophils. However, the effects of ivermectin on neutrophil spreading and morphology were reversed by L-NAME. L-NAME restored the area occupied by ivermectin-treated neutrophils (222 ± 10 μm^2^) to that of control neutrophils (246 ± 10 μm^2^) (Figure 3). The ability of L-NAME to restore morphology (Figure 2H) and the spreading (Figure 3) of ivermectin-treated neutrophils may indicate that the effect of ivermectin is due to the induction of NO production by neutrophils. This suggestion is consistent with evidence that ivermectin can induce NO production in rat neutrophils, which is responsible for killing microfilariae [45].

### 3.2. Reactive Oxygen Species Production by Neutrophils upon Adhesion to Fibronectin in the Presence of Ivermectin

**Figure 4:**
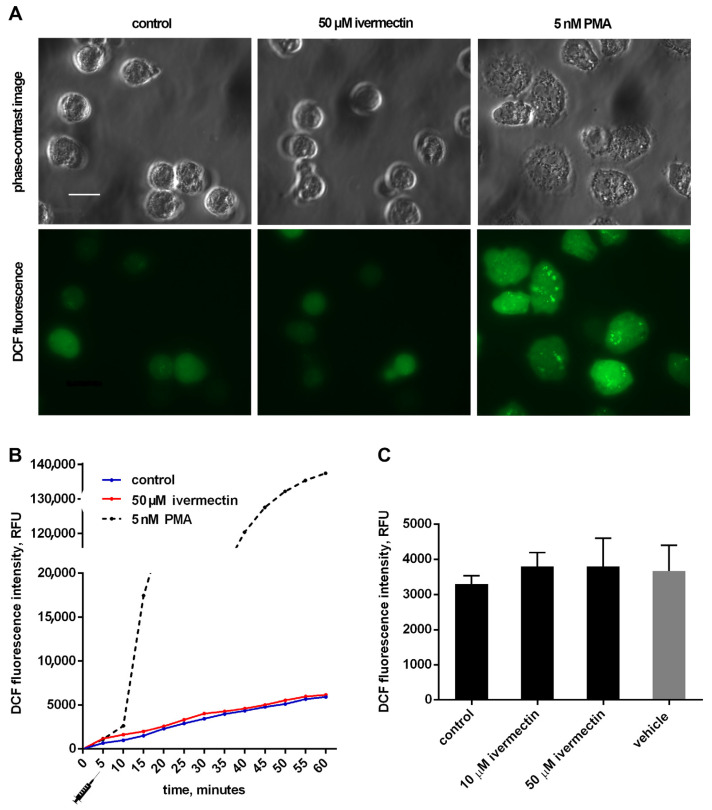
Effect of ivermectin on the formation of ROS during adhesion of neutrophils to fibronectin. H_2_DCF-DA-stained neutrophils were incubated in fibronectin-coated 96-well plates for 60 min at 37 °C in 5% CO_2_ under control conditions and in the presence of additives. (**A**) Phase-contrast and fluorescence images of H_2_DCF-DA-stained neutrophils that were attached to fibronectin for 25 min under control conditions or in the presence of ivermectin or PMA. (**B**) Representative DCF fluorescence kinetic curves for control, ivermectin- and PMA-treated neutrophils. Stimuli were added at the moment marked with the “syringe” icon. (**C**) DCF fluorescence intensity measured after 30 min incubation under control conditions and in the presence of ivermectin or vehicle (DMSO, 5 μL/mL). Values are means ± SEM from three independent experiments.

The effect of ivermectin on the intracellular production of ROS by neutrophils during adhesion to fibronectin was measured by the green fluorescence intensity of DCF, the oxidized product of H2DCF-DA. The results show that adhesion to fibronectin itself initiated the formation of ROS by neutrophils (Figure 4), which is consistent with data in the literature. Neutrophil adhesion to fibronectin is mediated by β-1 and β-2 receptors of the integrin family. It has been shown that the participation of β-2 integrin in the absence of neutrophil agonists can trigger the spreading of neutrophils, the formation of ROS, and the outflow of chloride ions [46]. The initiation of a signaling cascade, leading to the assembly of NADPH oxidase and ROS production, occurs also as a result of the binding of ß-1 integrin to a high-affinity binding site on fibronectin [47]. It should be noted that the formation of ROS induced by cell adhesion is insignificant in quantity. For comparison, the kinetic curve of ROS formation initiated under the same conditions by PMA, a powerful neutrophil activator, is presented [48]. The presence of ivermectin at a concentration of 10–50 µM did not change the ROS production by neutrophils during adhesion to fibronectin (Figure 4). The data show that ivermectin cannot initiate inflammation by stimulating the production of ROS by neutrophils.

### 3.3. Effect of Ivermectin on the Composition of Free Amino Acid Secretion by Neutrophils during Adhesion to Fibronectin

**Figure 5:**
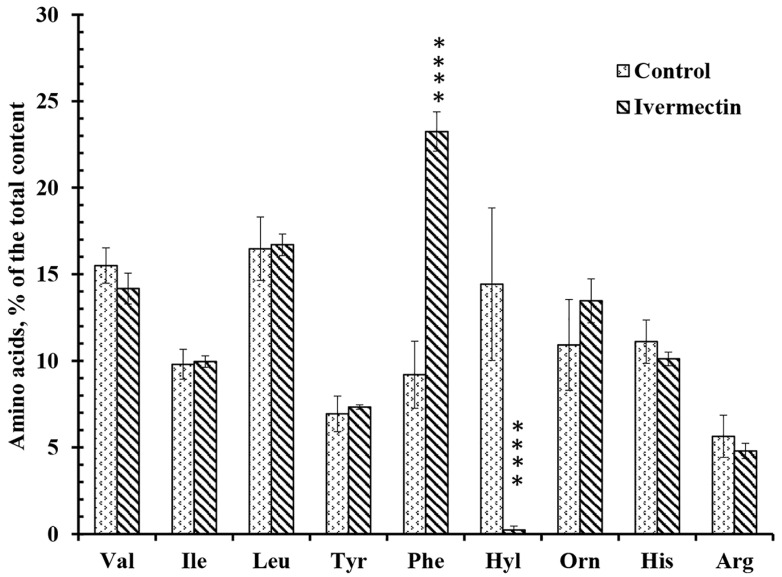
Effect of ivermectin on the free amino acid secretion by neutrophils during adhesion to fibronectin. Neutrophils were attached to fibronectin-coated substrates for 25 min under control conditions or in the presence of 50 μM ivermectin. The number of amino acids is presented as a percentage of the total content of free amino acids found (mean value ± standard error). Amino acid profiles were obtained by summing up the results of three independent experiments. ****—Significant differences compared to the value for the same amino acid under control conditions (*p* < 0.0001), as indicated by two-way ANOVA with Tukey’s multiple comparison test.

The secretion profiles of free amino acids by control neutrophils and neutrophils attached to fibronectin in the presence of ivermectin differed in the content of only two amino acids, hydroxylysine and phenylalanine (Figure 5). Ivermectin dramatically decreased the secretion of hydroxylysine but significantly stimulated the release of phenylalanine. To ensure that amino acids in the extracellular environment are not the result of cell destruction, neutrophils were stained with Trypan blue at the end of the experiment. The percentage of stained (dead) cells did not exceed 2% in any experimental group.

Our previous data have shown that the secretion of branched-chain, aromatic and positively charged amino acids (except hydroxylysine and phenylalanine) is a characteristic and stable property of neutrophils. The secretion of hydroxylysine is selectively stimulated by cell adhesion [15,16]. An increase in hydroxylysine release may be inhibited by minoxidil, a lysyl hydroxylase inhibitor, indicating lysyl hydroxylase activation upon adhesion [16]. A statistically significant increase in hydroxylysine release was observed when neutrophils attached to fibronectin in the presence of insulin or LPS [15,49]. In both cases, the stimulation of hydroxylysine release was selective, since both drugs had little effect on the secretion of other amino acids.

A number of agents that strongly inhibit the release of hydroxylysine have also been identified. These agents can be divided into two groups. Compounds of one group inhibited the release of hydroxylysine by neutrophils in combination with the stimulation of the secretion of tertiary granule components such as MMP, but not primary granule components. This group includes minoxidil, a lysyl hydroxylase (LH) inhibitor, and doxycycline, an inhibitor of matrix metalloproteinases (MMPs) [16]. These agents do not alter the secretion profile of amino acids, with the exception of hydroxylysine. Our data showed that minoxidil and doxycycline had similar effects on both free amino acid secretion and protein secretion during neutrophil adhesion, indicating a specific interaction between LH and MMP-9 in these processes.

Another group of drugs strongly suppressed the release of hydroxylysine, but also caused a statistically significant increase in the release of phenylalanine. This group includes microbial alkaloids that depolymerize the actin cytoskeleton, such as cytochalasin D, as well as the AKT 1/2 inhibitor or imipramine [15,16,50]. Cytochalasin D and other components of this group stimulated the secretion of serine proteases, such as cathepsin G, and other components of primary granules, but not the components of tertiary granules [28,29,35].

### 3.4. Effect of Ivermectin on the Composition of Protein Secretion by Neutrophils during Adhesion to Fibronectin

After the completion of the neutrophil adhesion process in the presence of test agents, samples of the extracellular medium were taken and neutrophils were stained with Trypan blue. The presence of stained cells did not exceed 2%, which indicates that proteins in the extracellular medium are not the result of cell death. Proteins were extracted and separated using SDS-PAGE. Silver staining of electrophoresis gels revealed numerous colored bands demonstrating that neutrophils secrete various proteins upon adhesion. However, in order to identify separated proteins using mass spectrometry, we must stain the protein bands with Coomassie brilliant blue. Coomassie brilliant blue stained only a limited number of major protein bands, but nevertheless made it possible to establish the protein secretion profiles that were quite stable and characteristic for tested agents. The profile included proteins that were significantly identified in three similar experiments.

**Figure 6:**
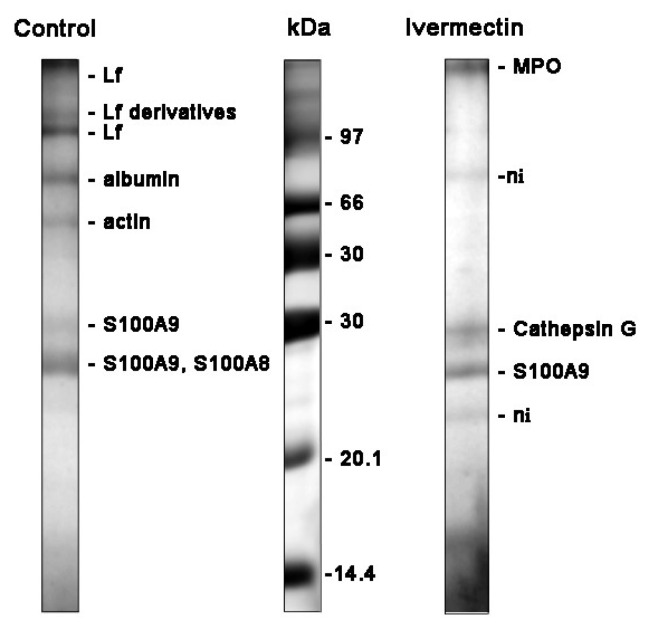
SDS-PAGE separation of proteins secreted by neutrophils upon adhesion to fibronectin. Human neutrophils were attached to fibronectin-coated substrates for 25 min incubation under control conditions or in the presence of 50 μM ivermectin. The extracellular medium was collected and the proteins were extracted. After concentration, the proteins were subjected to separation in 15% SDS-PAGE under non-reduced conditions. The gels were stained with Coomassie brilliant blue. The figures show typical protein profiles observed in three independent experiments.

The main proteins secreted by neutrophils during adhesion to fibronectin under control conditions were: lactoferrin (Lf) and its derivatives, predominantly localized in secondary granules; albumin, a component of secretory vesicles; as well as cytosolic proteins S100A9 and S100A8 (Figure 6, Table 1). Lf is an iron-binding glycoprotein with multiple functions in the body, including antimicrobial, anti-viral, anti-inflammatory, and anticancer actions [51,52]. Serum albumin, which is secreted from the secretory vesicles, enters them as a result of endocytosis during the stay of neutrophils in the blood. S100A9 is a pro-inflammatory protein that is abundantly expressed in the neutrophil cytosol and can cause the degranulation of specific, but not primary, neutrophil granules [53]. The high expression of S100A8 and S100A9 is discussed as an unfavorable prognostic factor for cancer patients [54].

The study of cellular secretion is carried out, as a rule, by measuring two or three proteins chosen by the experimenters, which may not be involved in secretion in a particular case. Our method of studying cellular secretion by establishing the protein composition of the extracellular environment allows us to establish a general picture of the secretion process and to identify the main proteins secreted in each specific case. However, proteins and proteases secreted by neutrophils interact with each other within 25 min of incubation. This makes the separation and identification of proteins difficult and many protein bands remain unidentified. The protein profile of neutrophil secretion during adhesion to fibronectin in the presence of ivermectin included primary granule components such as myeloperoxidase (MPO), cathepsin G, cytosolic S100A9 protein, and some non-identified proteins (marked ni) (Figure 6, Table 1). We included S100A9 in the list despite its protein match being non-significant since the corresponding protein band was observed in all experiments. The composition of secretion induced by ivermectin resembled that of secretion induced by cytochalasin D [35], which contained components of primary granules but not components of neutrophil tertiary granules, such as MMPs. MPO is one of the most abundantly expressed proteins in neutrophils. Aside from the involvement in the antimicrobial defense system, it contributes to neutrophil longevity, tissue inflammation and cancer [55]. Cathepsin G is one of the three serine proteases localized in the primary granules of neutrophils. The enzyme is involved in the clearance of pathogens, proteolytic modification of chemokines and cytokines, and shedding of cell surface receptors. The neutrophil serine proteases also play an important role in the development of cardiovascular diseases and chronic inflammation [36,56]. The high expression of S100A9 and S100A8, pro-inflammatory proteins, is associated with poor prognosis in patients with different types of cancer, including glioma or leukemia [57,58].

### 3.5. Is the Formation of Phenylalanine in the Extracellular Medium of Neutrophils upon Adhesion to Fibronectin Related to the Processes of Angiotensin Conversation?

In the previous paragraph, we noted that those compounds (minoxidil, doxycycline) that inhibited the release of hydroxylysine without affecting the secretion of other amino acids stimulated the secretion of tertiary granule proteins, such as MMPs. The results of our experiments showed that ivermectin belongs to another group of drugs that suppressed the release of hydroxylysine in combination with a statistically significant increase in the release of phenylalanine. Ivermectin, as well as cytochalasin D and other components of this group, stimulated the secretion of primary granule proteins, such as cathepsin G (Figure 5 and Figure 6, Table 1). Based on these results, the question arose whether the increase in the concentration of phenylalanine in our experiments is the result of the activity of cathepsin G or other serine proteases secreted by neutrophils in unidentified amounts (Figure 5 and Figure 6, Table 1). What substance could serve as a substrate for the release of phenylalanine in our experiments remains to be seen.

**Figure 7:**
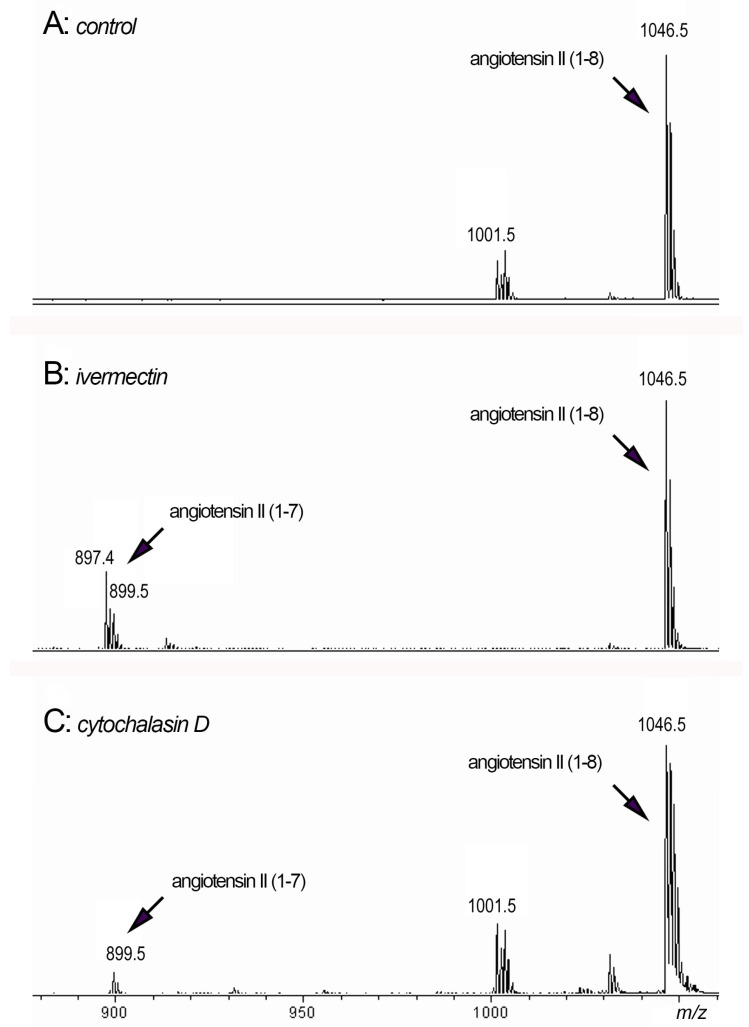
MALDI-TOF MS analysis of peptides in the extracellular environment of neutrophils after adhesion. Neutrophils were attached to fibronectin for 90 min in the presence of 50 μM angiotensin II (1–8) alone ((**A**): control) or in combination with 50 μM ivermectin ((**B**): ivermectin) or 10 μg/mL cytochalasin D ((**C**): cytochalasin D). Samples of the extracellular medium were taken and after removal of non-attached neutrophils by centrifugation, they were incubated for 120 min at 3 °C. Aliquots of the extracellular medium were taken and subjected to mass spectrometry.

We decided to conduct experiments with the addition of a possible substrate for the cleavage of phenylalanine, namely angiotensin II, to the extracellular environment of neutrophils. The data in the literature demonstrate that cathepsin G may rapidly convert angiotensin I to angiotensin II (1–8) [38]. Further inactivation of angiotensin II (1–8) may be a result of the cleavage of phenylalanine from the C-terminal resulting in the formation of angiotensin II (1–7) [59]. We studied whether the formation of angiotensin II (1–7) can occur upon neutrophil adhesion to fibronectin under control conditions or in the presence of ivermectin or cytochalasin D, which stimulated cathepsin G release, when exogenous angiotensin II (1–8) was added (Figure 7).

We observed the formation of an 899 Da peak corresponding to angiotensin II (1–7) when neutrophils were attached to a substrate in the presence of ivermectin (Figure 7B) or cytochalasin D (Figure 7C) in combination with angiotensin II (1–8) (peak 1046.5 Da). The data showed that neutrophil secretion induced by ivermectin or cytochalasin D could, in principle, cleave phenylalanine from angiotensin II, thereby inactivating this physiologically active compound. In addition to cathepsin G, other proteases secreted by neutrophils in trace amounts may also be involved in the process of the cleavage of phenylalanine.

## 4. Discussion

The anthelmintic drug ivermectin is a synthetic derivative based on the microbial alkaloid avermectin isolated from a strain of the soil actinomycete Streptomyces avermitili [60]. Recent studies have also revealed the antiviral activity of ivermectin associated with the ability to inhibit flavivirus replication by targeting the NS3 helicase [61], block the importin alpha/beta-mediated nuclear transport of viral proteins, capable of inhibiting the replication of HIV-1 and dengue virus [62], and inhibit SARS-CoV-2 replication in vitro [63]. Currently, the possibility of using ivermectin for the prevention and treatment of inflammatory processes induced by viral diseases [8,9,10,11,12,13], as well as for the treatment of cancer patients [14], is being studied. We assumed that the therapeutic effect of ivermectin is associated not only with antiviral activity, but also with its effect on human innate immunity, namely, with its effect on neutrophil function.

Neutrophils, the first line of antimicrobial defense of innate immunity, normally move in the bloodstream, temporarily attaching to the vascular lining endothelium with selectin family receptors. When neutrophils enter the focus of infection, selectins are shed under the action of chemoattractants and neutrophils are attached to the endothelium by receptors of the integrin family. The integrin-dependent adhesion of neutrophils is accompanied by the secretion of aggressive bactericidal agents that cause the inflammation of blood vessels and surrounding tissues [1,64]. Inflammation induced by recruited neutrophils is the cause of progressive lung damage in cystic fibrosis or chronic obstructive pulmonary disease [3]. A high neutrophil-to-lymphocyte ratio and extensive neutrophil infiltration into the pulmonary capillaries and into the alveolar space has been observed in pneumonia, progressing to acute respiratory distress syndrome (SARS), including COVID-19 [65,66]. Comparison of blood transcripts from COVID-19 patients and healthy donors showed that signatures associated with neutrophil activation were markedly enriched in patients with severe disease [67].

We studied the effect of ivermectin on integrin-dependent neutrophil adhesion to fibronectin and the associated secretion. Our data indicate that ivermectin cannot stimulate tissue inflammation through the initiation of oxidative stress in neutrophils, since it did not affect the attachment of neutrophils to fibronectin (Figure 1) or the production of ROS by neutrophils during adhesion (Figure 4). However, ivermectin can inhibit neutrophil-induced inflammation by inhibiting neutrophil invasion into tissues. Our previous data showed that minoxidil, an LH inhibitor, suppresses the release of hydroxylysine by neutrophils upon adhesion. [16]. The inhibition of hydroxylysine release by ivermectin (Figure 5) indicates a decrease in LH activity, but it remains to be seen whether ivermectin inhibits intracellular LH or LH secreted by neutrophils into the extracellular environment. The LH activity plays an important role in the ability of tumor cells to invade and metastasize through the modification of the extracellular matrix. A growing number of articles present increased LH expression as a prognostic marker associated with poor prognosis in patients with osteosarcoma [68], glioblastoma [27], oral squamous cell carcinoma [25,69], and other cancer diseases. Moreover, neutrophils that were attached to fibronectin in the presence of ivermectin did not secrete MMPs (Figure 6, Table 1). The inhibition of MMP activity by doxycycline or MMP-9 deficiency per se inhibits leukocyte recruitment [33,34]. LH and MMP closely interact during the reorganization of the extracellular matrix, which is necessary for cell migration and adhesion, both in the extracellular environment and during immobilization on the cell surface [70].The ability of ivermectin to inhibit cell migration has been demonstrated in wound healing experiments on HeLa cells [71]. Ivermectin is currently being studied as an anti-cancer agent that can inhibit tumor cell invasion into tissues leading to the formation of metastases [14].

Cathepsin G, secreted by neutrophils upon adhesion to fibronectin (Figure 6, Table 1), may contribute to neutrophilic inflammation in a variety of ways, including the destruction of neutrophil surface receptors [37] and the increased hydrolysis of the extracellular environment [36]. Cathepsin G can also influence neutrophilic inflammation through the physiological renin–angiotensin–aldosterone pathway, which regulates the synthesis and bioavailability of NO [72], a physiological mediator that prevents the integrin-dependent adhesion of leukocytes [73]. Angiotensin II uncouples NO synthase and stimulates the formation of NO-binding ROS, thereby reducing NO bioavailability [74]. In our experiments, the NO synthase inhibitor L-NAME eliminated changes in the morphology and spreading of neutrophils induced by ivermectin (Figure 2 and Figure 3), which indicates the involvement of NO in the effect of ivermectin. Angiotensin II is formed from angiotensin I under the action of angiotensin-converting factor (ACE). Soluble cathepsin G in in vitro experiments and cathepsin G, which appears on the surface of isolated neutrophils as a result of activation, have been shown to rapidly convert angiotensin I to angiotensin II [38,40]. At the same time, cathepsin G was shown to rapidly degrade angiotensin II as a result of the cleavage of angiotensin II between four and five residues [39]. These data indicate that cathepsin G could provide an alternative to ACE mechanism for the rapid local release of angiotensin II at the site of inflammation, as well as provide a mechanism to limit its action prior to it escaping into systemic circulation.

The physiological inactivation of pro-inflammatory angiotensin II (1–8) to anti-inflammatory angiotensin II (1–7) is carried out by a zinc metalloproteinase angiotensin converting enzyme 2 (ACE2) via the cleavage of the C-terminal phenylalanine [59]. ACE2 has been found to have an affinity for the S-glycoproteins of some coronaviruses [75,76,77], and is thus the entry point of the virus into the cell. Coronavirus infection may, by suppressing ACE2, lead to a toxic excess accumulation of angiotensin II, which causes acute respiratory distress syndrome or pulmonary edema. We observed the formation of angiotensin II (1–7) in experiments when neutrophils adhered to fibronectin in the presence of ivermectin or cytochalasin D, which stimulated cathepsin G release, and exogenously added angiotensin II (1–8) (Figure 7). It may indicate that ivermectin-induced neutrophil secretion including cathepsin G could, in principle, inactivate angiotensin II by the cleavage of C-terminal phenylalanine, thus forming a local alternative for ACE2 that may be suppressed upon coronavirus disease.

In conclusion, we hypothesize that the suppression of neutrophil invasion into tissues by ivermectin may have a therapeutic effect on the development of neutrophil-induced inflammation in cystic fibrosis, chronic obstructive pulmonary disease, and other pulmonary pathologies, including coronavirus disease. On the other hand, ivermectin induces the release of cathepsin G by neutrophils upon adhesion. As was show in in vitro experiments with separated enzyme or isolated neutrophils, cathepsin G may be involved in both the formation of pro-inflammatory angiotensin II and its inactivation by cleavage of the molecule between the fourth and fifth amino acid residues or by cleavage of the C-terminal phenylalanine (Figure 7). We can suggest that the effect of cathepsin G secreted by neutrophils in response to ivermectin would depend on the physiological concentration of angiotensin I and angiotensin II and have a local character.

Inflammatory processes caused by neutrophils occur both during infection and in the absence of infection in metabolic disorders, including cystic fibrosis or pneumonia provoked by viral diseases, including COVID-19. In these cases, inflammation occurs in the absence of infection, so the use of antibiotics becomes useless. A promising direction in the search for a therapy for neutrophilic inflammation is the identification of physiological pathways that regulate the mechanisms of invasion and secretion of neutrophils, and ways to influence these pathways. In this regard, components and inhibitors of the physiological system of angiotensin I–angiotensin II–aldosterone conversion, which can regulate neutrophil adhesion and secretion, acting through the synthesis and bioavailability of nitric oxide (NO), are of undoubted interest.

## Figures and Tables

**Table 1:** List of proteins secreted by neutrophils during adhesion to fibronectin. Neutrophils were attached to fibronectin for 25 min under control conditions or in the presence of 50 μM ivermectin. Proteins were separated by SDS-PAGE and identified by mass spectrometric analysis. Protein matches were considered significant (*p* < 0.05) if the score was >68. Proteins identified in three analogous experiments were included in the list.

Treatment	Protein Name		Peptides Matched/ Total	Coverage %	MOWSE Score
control	TRFL_HUMAN	LF	18/28	23	141
	ALBU_HUMAN	albumin	23/56	49	115
	AKTB_HUMAN	actin	2/74	27	72
	S10A9_HUMAN	S100-A9	7/24	60	96
	S10A8_HUMAN	S100-A8	6/24	45	75
ivermectin	PERM_HUMAN	MPO	13/38	14	79
	CATG_HUMAN	cathepsin G	8/21	20	72
	S10A9_HUMAN	S100-A9	4/50	33	30
